# Remnant cholesterol is an effective biomarker for predicting survival in patients with breast cancer

**DOI:** 10.1186/s12937-024-00951-3

**Published:** 2024-04-22

**Authors:** Jinyu Shi, Tong Liu, Chenan Liu, Heyang Zhang, Guotian Ruan, Hailun Xie, Shiqi Lin, Xin Zheng, Yue Chen, Qi Zhang, Xiaowei Zhang, Xiangrui Li, Xiaoyue Liu, Li Deng, Han-Ping Shi

**Affiliations:** 1 Department of Gastrointestinal Surgery, Department of Clinical Nutrition, Beijing Shijitan Hospital, Capital Medical University, Beijing, 100038 China; 2 National Clinical Research Center for Geriatric Diseases, Xuanwu Hospital, Capital Medical University, Beijing, 100053 China; 3 Laboratory for Clinical Medicine, Capital Medical University, Beijing, 100053 China; 4 Key Laboratory of Cancer FSMP for State Market Regulation, Beijing, 100038 China; 5 Department of Genetics, Yale School of Medicine, New Haven, CT 06510 USA

**Keywords:** Remnant cholesterol, Breast cancer, Prognosis, Biomarker

## Abstract

**Background:**

Breast cancer is the most common malignancy in women worldwide. The relationship between remnant cholesterol (RC) and the prognosis of patients with breast cancer has not been clearly reported. This study investigated the prognostic value of RC in predicting mortality in patients with breast cancer.

**Methods:**

This study prospectively analysed 709 women patients with breast cancer from the Investigation on Nutrition Status and Clinical Outcome of Common Cancers (INSCOC) project. Restricted cubic splines were used to analyse the dose-response relationship between RC and breast cancer mortality. The Kaplan–Meier method was used to evaluate the overall survival of patients with breast cancer. A Cox regression analyses was performed to assess the independent association between RC and breast cancer mortality. Inverse probability of treatment weighting (IPTW) using the propensity score was used to reduce confounding. Sensitivity analysis was performed after excluding patients with underlying diseases and survival times shorter than one year.

**Results:**

A linear dose-response relationship was identified between RC and the risk of all-cause mortality in patients with breast cancer (*p* = 0.036). Kaplan–Meier survival analysis and log-rank test showed that patients with high RC levels had poorer survival than those with low RC levels (*p* = 0.007). Univariate and multivariate Cox regression analyses showed that RC was an independent risk factor for mortality in women patients with breast cancer. IPTW-adjusted analyses and sensitivity analyses showed that CR remained a prognostic factor.

**Conclusions:**

RC is an independent risk factor for the prognosis of patients with breast cancer, and patients with higher RC levels have poorer survival.

**Supplementary Information:**

The online version contains supplementary material available at 10.1186/s12937-024-00951-3.

## Background

Breast cancer is the most common malignant tumour in women, and its case fatality rate ranks second among women tumours, posing a major threat to women’s health and quality of life [1]. Despite remarkable advances in preventing, diagnosing, and treating breast cancer, its survival and prognosis remain challenging. Therefore, identifying effective biomarkers is important for survival prediction and individualised treatment of patients with breast cancer.

Previous studies have shown that high cholesterol and triglyceride levels are associated with cancer development, recurrence, and worse survival [2–6]. This finding may be attributed to the association between high blood lipid levels and factors such as chronic inflammation, obesity, and metabolic syndrome, all of which can influence tumour growth and progression [7–9]. In addition, an increasing number of studies have demonstrated a relationship between cholesterol and cancer, including an important role in tumour cell proliferation, angiogenesis, immune regulation, and other processes [10, 11]. In recent years, with the widespread use of statins, the focus of research has gradually shifted to triglyceride-rich lipoproteins. Remnant cholesterol (RC) is the lipoprotein remnant at different stages of triacylglycerol degradation or lipoprotein conversion, and is the cholesterol content of triglyceride-rich lipoproteins.

RC is a unique form of cholesterol that has been attracting increasing attention. RC refers to cholesterol components other than high-density lipoproteins (HDL) and low-density lipoproteins (LDL), including very low–density lipoproteins (VLDL), intermediate-density lipoproteins (IDL), and chylomicron residues [12–14]. The role of RC molecules in the tumour microenvironment has received increasing attention. However, the relationship between RC and the prognosis of patients with breast cancer has not been clearly reported, and further studies are needed to confirm this result.

Therefore, this study investigated the relationship between RC and the mortality of patients with breast cancer in a large cohort study to provide more accurate guidance for individualised treatment and survival prediction of patients with breast cancer and to improve their survival rate and quality of life.

## Methods

### Study population

This study was based on the Investigation on Nutrition Status and its Clinical Outcome of Common Cancers (INSCOC) project, conducted in more than 40 hospitals in China and registered at chictr.org.cn (registration number: ChiCTR1800020329). This project prospectively collected the clinical data of patients with cancer. This study screened 1194 women patients with breast cancer who visited the clinic between June 2012 and December 2019, 485 patients with incomplete clinical or survival data were excluded, and 709 patients were included in the final data analysis. All patients were > 18 years of age, had a pathological diagnosis of cancer, and had complete clinical data and follow-up information. The flowchart is shown in Figure S1. Written informed consent was obtained from all the enrolled patients. This study was approved by the ethics committees of all the participating hospitals and was performed in accordance with the guidelines of the Declaration of Helsinki.

### Baseline data collection

General clinical characteristics and laboratory biochemical indicators were collected for all enrolled patients within 48 h after admission, including age, height, weight, TNM stage, diabetes, hypertension, coronary heart disease, family history of tumour, smoking history, drinking history, haemoglobin, white blood cells, neutrophils, lymphocytes, platelets, cholesterol, triglycerides, HDL, LDL, blood glucose, total bilirubin, direct bilirubin, aspartate aminotransferase, glutamate aminotransferase, total protein, albumin, creatinine, and blood urea nitrogen levels. The same protocol and reference ranges are used for laboratory testing nationwide. All these clinical characteristics and laboratory biochemical indicators were collected when first included in the INSCOC project.

TNM stage of tumour was performed according to the 8th edition of the American Joint Committee on Cancer guidelines. Body mass index (BMI) was calculated as weight (kg) divided by height squared (m^2^). The patients were divided into three groups based on BMI levels: underweight (< 18.5 kg/m^2^), normal weight (18.5–24.0 kg/m^2^), and overweight (≥ 24 kg/m^2^). Patients were divided into two groups (< 50 years and ≥ 50 years) based on baseline age.

RC is defined as cholesterol content other than LDL and HDL, including VLDL, IDL, and chylomicrons. The RC was calculated as follows: RC = cholesterol – LDL – HDL [14].

### Follow-up and outcomes

The included patients were followed up continuously through hospitalisation, outpatient clinic visits, or telephone calls, and survival data were recorded. The primary outcome was overall survival (OS), defined as the period from pathological diagnosis to death or the last follow-up.

### Statistical analysis

Measurement data were expressed as the mean ± standard deviation or median (interquartile range). The differences of RC between the groups were evaluated using the Mann–Whitney test. Restricted cubic splines (RCS) were used to explore the dose-response association between RC and mortality. We performed a dichotomous classification of continuous variables based on the optimal cut-off values calculated using maximal rank statistics. Kaplan–Meier curves and log-rank tests were used to compare the survival rates. The proportional hazard assumption was checked using Schoenfeld residual test. Univariate and multivariate Cox regression models were used to calculate hazard ratios (HR) and 95% confidence intervals for RC in the two models (crude and adjusted models). The crude model was not adjusted for the covariates. The model was adjusted for age, TNM stage, BMI, diabetes, hypertension, coronary heart disease, smoking, drinking, and family history of tumours. Inverse Probability of Treatment Weighting (IPTW)-adjusted Kaplan-Meier survival curves and IPTW-adjusted COX regression analyses were used to reduce confounding. To confirm the stability of the correlation between RC and OS, sensitivity analysis was performed after excluding patients with underlying diseases (diabetes, hypertension, and coronary heart disease) and those who died within 1 year. Statistical significance was set at *p* < 0.05. All statistical analyses were performed using R4.2.1 software.

## Results

### Patient characteristics

In total, 709 women breast cancers were included in this study. The median age of patients was 52 years (IQR, 46.00–61.00 years), the median BMI was 23.88 kg/m^2^ (IQR, 21.66–26.29 kg/m^2^), and the median RC was 0.58 mmol/L (IQR, 0.32–0.94). According to the TNM stage, 19.3% (137/709), 30.5% (216/709), 16.6% (118/709), and 33.6% (238/709) of patients were in stages I, II, III, and IV, respectively; 64 cases (9.0%) had diabetes, 110 (15.5%) had hypertension, and 27 (3.8%) were undergoing chronic haemodialysis. In addition, 15.5% of the patients died during the follow-up period, and the median OS was 32.23 months (IQR, 18.1–57.4). The baseline characteristics of the patients are summarised in Table S1.

Figure S2 shows that women ≥ 50 years had higher RC levels than women < 50 years. With the increase of BMI, the RC level of the patients gradually increased. Interestingly, there was no statistically significant difference between tumour stage and patients’ RC level.

### Optimal cut-off value of RC and Kaplan–Meier curve

**Fig. 1:**
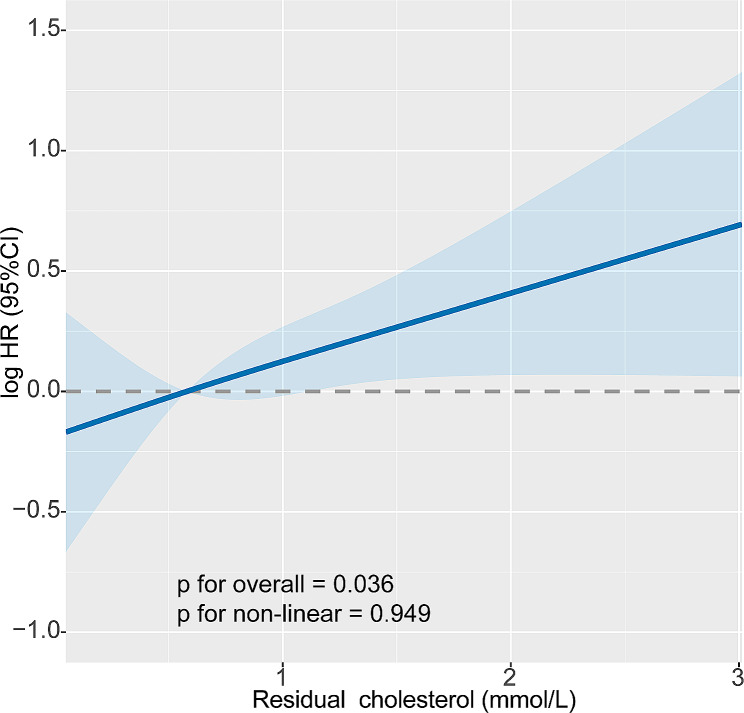
The relationship between remnant cholesterol and overall survival in patients with breast cancer. Note: Adjusted for age, TNM stage, BMI, diabetes, hypertension, coronary heart disease, smoking, drinking, family history of tumour

The RCS was used to evaluate the relationship between RC and mortality in women patients with breast cancer (Fig. 1). We observed a linear dose-response relationship between RC and mortality (*p* = 0.036). Moreover, we separately assessed the relationship between total cholesterol, HDL, LDL levels and survival. Figure S3 shows a U-shaped dose-response relationship between total cholesterol and patient survival, indicating that patients with higher or lower total cholesterol levels have poorer prognosis. There was no significant difference between HDL, LDL and survival.

**Fig. 2:**
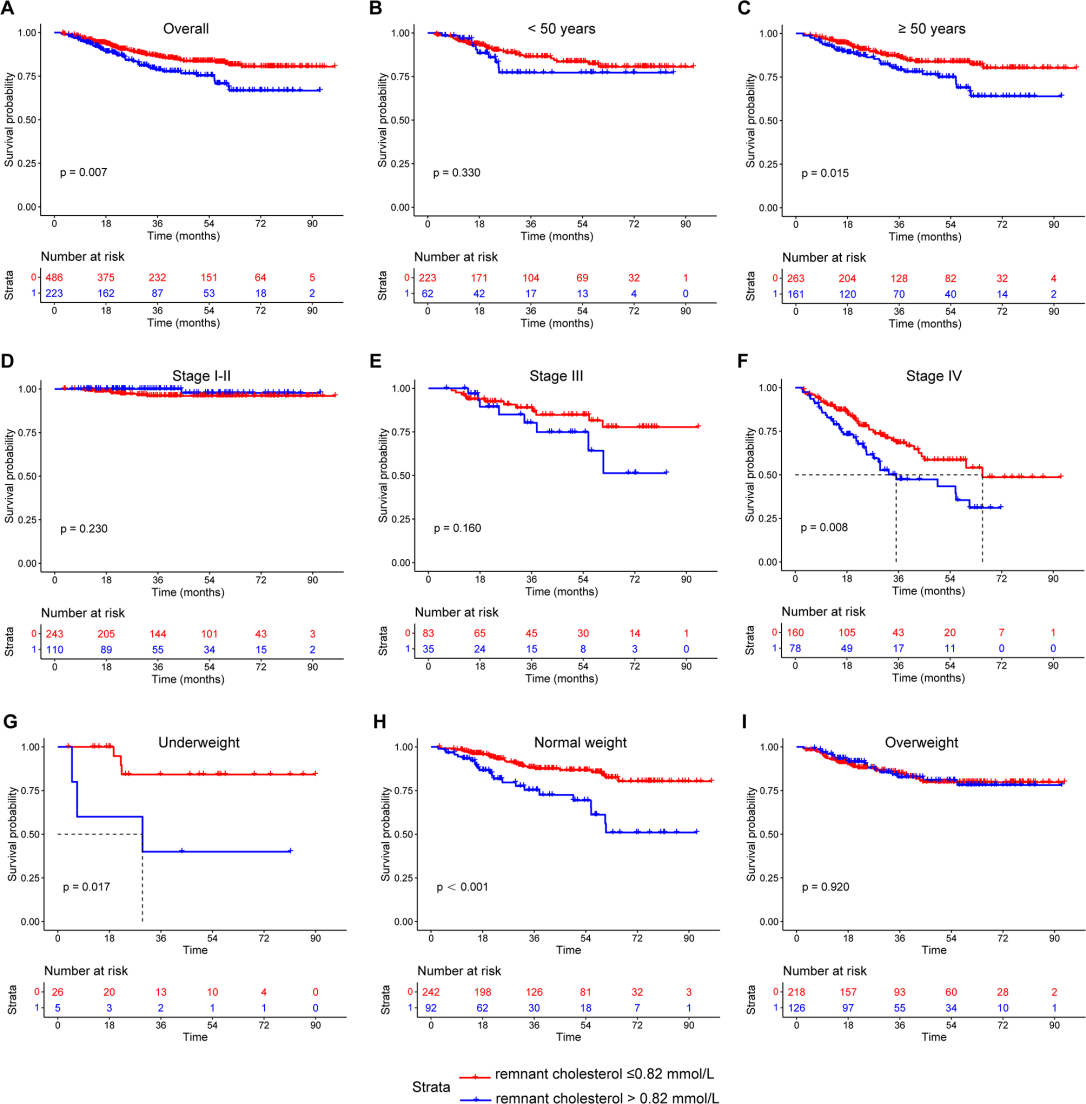
The Kaplan–Meier curves of breast cancer patients with low or high remnant cholesterol. (**A**) total population; (**B**) patients < 50 years; (**C**) patients ≥ 50 years; (**D**) stage I–II patients; (**E**) stage III patients; (**F**) stage IV patients; (**G**) underweight patients; (**H**) normal weight patients; (**I**) overweight patients

We then used the maximal rank statistics for the binary classification of RC and obtained an optimal cut-off value of 0.82 mmol/L (Figure S4). The patients were then divided into a high RC group (*n* = 223) and a low RC group (*n* = 486) based on the optimal cut-off value. Kaplan–Meier survival curves were established for both groups. As shown in Fig. 2, the survival of patients in the high-RC group was significantly shorter than that of patients in the low-RC group (*p* = 0.007). In addition, we established Kaplan–Meier survival curves for different ages (< 50 years and ≥ 50 years), tumour stages, and BMI levels. The analysis showed that patients with high RC levels had poorer survival inpatients ≥ 50 years, stage IV, underweight, and normal-weight patients. No significant relationship was found between RC and survival inpatients < 50 years, stage I-II, stage III, and overweight patients. The result of Kaplan–Meier survival curves after IPTW correction were similar to those without IPTW in both the total population and subgroups (Figure S5).

### Prognostic role of RC

The proportional hazards assumption was verified by the global test of Schoenfeld residuals (*p* = 0.352). Univariate and multivariate Cox regression analyses revealed that RC was significantly associated with mortality in patients with breast cancer (Table 1). Patients with high RC had a greater mortality risk than those with low RC (HR = 1.64, 95%CI = 1.11–2.45, *p* = 0.014). When RC was divided into tertiles (T1 group: RC < 0.41, T2 group: 0.41 ≤ RC < 0.80, T3 group: RC ≥ 0.80), the risk of death in the T2 group showed no significant difference compared to that in the T1 group (HR = 1.22, 95%CI = 0.75–1.99, *p* = 0.431). However, the risk of death in the T3 group was significantly higher than that in the T1 group (HR = 1.60, *p* = 0.048). We performed IPTW-adjusted Cox regression analyses to assess the prognostic role of CR while accounting for potential confounding factors and residual correlations (Table S2). The results of this analyses were found to be similar to those obtained without IPTW, indicating that CR remains a prognostic factor even after adjusting for these potential correlations.

**Table 1:** The univariate and multivariate cox analyses for the relationship between remnant cholesterol and all-cause mortality in patients with breast cancer

	Crude model		Adjusted model	
	HR (95%CI)	p	HR (95%CI)	p
Continuous (*n* = 709)	1.33 (1.06,1.68)	0.015	1.27 (1.01,1.59)	0.037
Cutoff value				
C1 (≤ 0.82, *n* = 486)	Ref		Ref	
C2 (> 0.82, *n* = 223)	1.68 (1.15,2.46)	0.007	1.64 (1.11,2.45)	0.014
Tertiles				
T1 (< 0.41, *n* = 237)	Ref		Ref	
T2 (< 0.80, *n* = 236)	1.10 (0.67,1.78)	0.710	1.22 (0.75,1.99)	0.431
T3 (≥ 0.80, *n* = 236)	1.59 (1.01,2.5)	0.045	1.60 (1.01,2.56)	0.048
p for trend		0.042		0.048

Notes: Crude model: no adjusted

Adjusted model: adjusted for age, TNM stage, BMI, diabetes, hypertension, coronary heart disease, smoking, drinking, family history of tumour

In addition, we assessed the Harrell’s C index for the fully adjusted model and compared it to the Harrell’s C index with the fully adjusted model without RC (Table S3). We found that the Harrell’s C index for the fully adjusted model was greater than that for the model without RC (C index: 0.80, 0.76, respectively), and the difference was statistically significant (*p* = 0.031).

### Sensitivity analyses and stratified analyses

After excluding patients with underlying diseases (including diabetes, hypertension, and coronary heart disease), univariate and multivariate Cox regression analyses revealed that RC was an independent risk factor for survival in patients with breast cancer (Table 2). Furthermore, we obtained similar results after excluding patients who survived < 1 year (Table 2).

**Table 2:** The sensitivity analyses of the relationship between remnant cholesterol and mortality by excluding patients with underlying diseases (diabetes, hypertension and coronary heart disease) and excluding patients with short-term deaths (1-year)

excluding patients with underlying diseases				
	Crude model		Adjusted model	
	HR (95%CI)	p	HR (95%CI)	p
Continuous (*n* = 550)	1.45 (1.16,1.82)	0.001	1.38 (1.1,1.71)	0.004
Cutoff value				
C1 (≤ 0.82, *n* = 391)	Ref		Ref	
C2 (> 0.82, *n* = 159)	1.94 (1.27,2.99)	0.002	1.81 (1.15,2.82)	0.01
Tertiles				
T1 (< 0.41, *n* = 202)	Ref		Ref	
T2 (< 0.80, *n* = 180)	1.42 (0.82,2.45)	0.210	1.56 (0.91,2.7)	0.109
T3 (≥ 0.80, *n* = 168)	2.09 (1.24,3.52)	0.005	1.93 (1.13,3.28)	0.016
p for trend		0.005		0.015

**excluding patients with short-term deaths (1-year)**				
Continuous (*n* = 648)	1.56 (1.2,2.02)	0.001	1.51 (1.18,1.93)	0.001
Cutoff value				
C1 (≤ 0.82, *n* = 450)	Ref		Ref	
C2 (> 0.82, *n* = 198)	1.84 (1.17,2.9)	0.009	1.97 (1.22,3.18)	0.006
Tertiles				
T1 (< 0.41, *n* = 218)	Ref		Ref	
T2 (< 0.80, *n* = 220)	1.31 (0.72,2.37)	0.377	1.35 (0.74,2.48)	0.324
T3 (≥ 0.80, *n* = 210)	1.89 (1.08,3.31)	0.025	1.96 (1.1,3.49)	0.023
p for trend		0.023		0.022

Notes: Crude model: no adjusted

Adjusted model: adjusted for age, TNM stage, BMI, diabetes, hypertension, coronary heart disease, smoking, drinking, family history of tumour

**Fig. 3:**
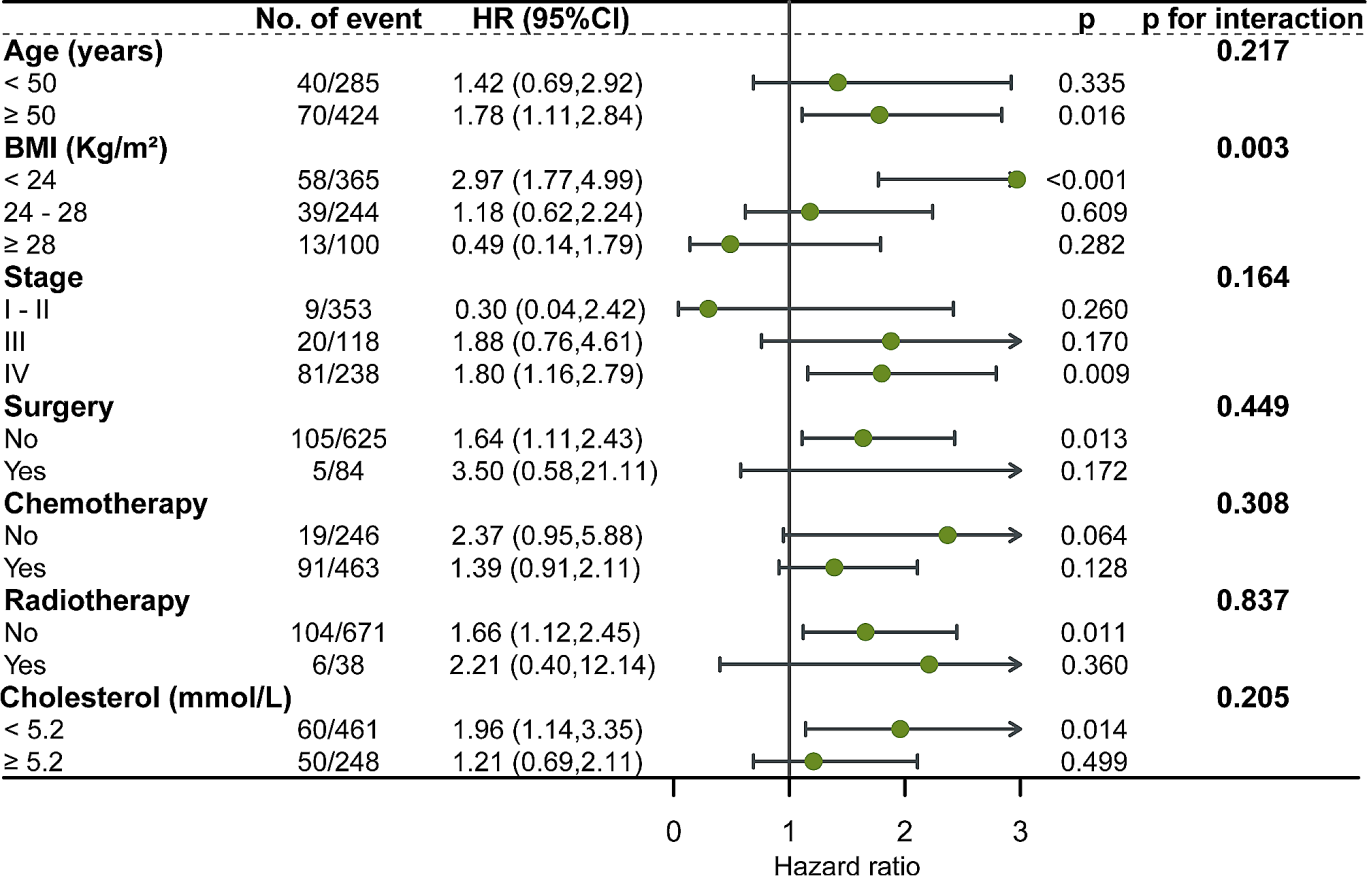
Subgroup analyses of the relationship between remnant cholesterol (as a dichotomous variable) and mortality in patients with breast cancer

Stratified analyses showed the relationship between RC and mortality in difference subgroups, including age (< 50 years vs.≥ 50 years), BMI (underweight vs. normal weight vs. overweight), tumour stage, surgery, chemotherapy, radiotherapy, and total cholesterol covariates (Fig. 3). As shown in Fig. 3, BMI interacts with residual cholesterol. The other variables had no significant interaction.

## Discussion

This study identified the relationship between RC levels and prognosis in women with breast cancer. We found that higher RC levels were associated with a higher risk of death. In addition, we performed IPTW-adjusted Kaplan-Meier analyses and IPTW-adjusted Cox regression analyses to evaluate the prognostic role of CR, and the results showed that CR was still a prognostic factor. After excluding patients with underlying diseases or those with a survival period < 1 year, RC can stably predict the risk of death in patients with breast cancer.

Breast cancer is a complex disease, and its occurrence and development involve the interaction of multiple genetic, hormonal, and lifestyle factors [15]. Studies have shown that high cholesterol levels increase the risk of breast, colorectal, and other cancers [3, 16, 17]. In addition, studies have shown that abnormal cholesterol levels are associated with the degree of malignancy and a poor prognosis in patients [3]. Cholesterol is a lipid with several important functions in the body, including the construction of cell membranes, hormone synthesis, and absorption of fat-soluble vitamins in the digestive system [18]. Dysregulation of cholesterol homeostasis is a feature of many diseases, including pathological conditions, tumours, metabolic disorders, and atherosclerosis [19].

Studies have shown that cholesterol increases the proliferation of oestrogen receptor–positive breast cancer cells via the metabolite 27-hydroxycholesterol, a molecule with selective oestrogen receptor modulatory activity [5, 10], whose levels are elevated in breast tumour biopsies compared to those in normal breast tissue [20]. In addition, dyslipidaemia leads to increased cholesterol content in cell membranes, affecting membrane fluidity and subsequent signalling [21]. Most current research has focused on total cholesterol, HDL, LDL, and triglycerides. RC is a lipoprotein residue involved in different stages of triacylglycerol degradation or lipoprotein conversion [14]. This value is the sum of the cholesterol levels in all triacylglycerol-rich lipoproteins, and its concentration is related to the degree of obesity and genetic polymorphisms and is highly correlated. Recently, with the application of statins, the focus of research has gradually shifted towards triacylglycerol-rich lipoproteins.

Previous studies on RC have focused mostly on cardiovascular and cerebrovascular diseases, diabetes, and other metabolic disorders [22, 23]. This study is the first to comprehensively evaluate the relationship between RC and survival in women breast cancer. The RCS and COX regression analyses suggested that RC was significantly and positively correlated with the risk of death in patients with breast cancer. Previous studies have reported that breast cancer is closely correlated with obesity, diabetes, hypertension, and other metabolic diseases [24]. Metabolic disorders may lead to imbalances in the synthesis and degradation of fatty acids, abnormal oestrogen synthesis and metabolism, and abnormal energy metabolism pathways in cells, thereby affecting the development and prognosis of breast cancer [21, 25]. In addition, metabolic disorders in the body can lead to insulin resistance, which, in turn, affects the development of breast cancer by promoting the insulin-like growth factor pathway [9].

Although the results of this study support the prognostic role of RC in women patients with breast cancer, the exact mechanism by which high RC levels are associated with poor survival outcomes remains uncertain. Our findings suggest that women aged ≥ 50 years have greater RC levels, which may be related to changes in hormone levels before and after menopause. Cholesterol is a precursor of androgens and oestrogens, which are closely associated with the occurrence and development of breast cancer [26]. Cholesterol metabolism interacts with hormones, affecting breast cancer cells [27, 28].

Patients with a higher BMI had greater RC levels. Some studies have shown that obesity may affect cholesterol synthesis, metabolism, and transport pathways [7, 8]. In obesity, the adipose tissue produces inflammatory factors, resulting in a chronic inflammatory state [29]. This inflammatory state may promote the development of breast cancer by activating pathways, including cell proliferation, invasion, and angiogenesis. In addition, abnormal cholesterol levels are strongly linked to metabolic syndrome, which is often associated with obesity [7]. Metabolic syndrome includes high blood pressure, high blood sugar, high cholesterol, and central obesity, among others, and the interaction of these factors may increase the risk of cardiovascular disease and diabetes [30]. Patients with higher tumour stages tended to have higher RC levels, possibly related to their overall metabolic disorders.

The relationship between breast cancer and cholesterol metabolism is complex, diverse, and involves multiple molecular pathways and biological processes. An in-depth study of the interaction between breast cancer and cholesterol metabolism will help better understand the mechanism, development process, and formulation of treatment strategies for breast cancer. It also provides a potential direction for identifying new therapeutic targets and intervention pathways.

Breast cancer is a highly heterogeneous disease, patients may have different biological characteristics and metabolic states. Monitoring RC levels can detect potentially high-risk patients earlier to enable more active interventions and reduce the risk of disease recurrence and progression, thereby improving the pertinence and effectiveness of treatment and realising individualised treatment. In addition, RC detection is non-invasive, simple, objective, and easy to implement. During the diagnosis and treatment of patients with breast cancer, repeated and dynamic assessments can be performed to avoid ineffective antitumour treatment and limited survival benefits and to maximise cost-effectiveness.

This study had several limitations. First, the study population comprised only Chinese patients. Considering the racial differences, the results of this study need to be verified in a larger study with a different population. It is worth noting that the proportion of people who received radiotherapy in this study was low, which may be due to the large differences in the utilization of radiotherapy for different tumour types in China. Head and neck cancers are more commonly treated with radiation; however, other types, such as breast cancer, tend to have lower rates of use. Second, the RC level was assessed only at baseline, and the relationship between its dynamic changes and patient prognosis could not be determined. Third, this study lacks data on molecular typing of patients, endocrine therapy and anti-HER-2 therapy, we plan to conduct further data improvement in the future. Forth, the database did not stratify surgery as upfront or after neoadjuvant, or chemotherapy as neoadjuvant or adjuvant. This is relevant since the change from clinical to pathological staging can impact the prognostic value obtained in adjusted analyses. Furthermore, the dataset lacks information on breast cancer mortality and mortality due to causes other than breast cancer in our study. We plan to improve the data collection in the future. In addition, we need to further explore the molecular mechanisms underlying RC metabolism to better screen for risk groups with poor prognoses.

## Conclusion

In summary, this study evaluated the predictive value of RC for the prognosis of women with breast cancer. The analysis showed that RC was an independent risk factor for the prognosis of patients with breast cancer. As an objective, convenient, non-invasive, and reproducible indicator, RC has the potential to be widely used in clinical practice. Measuring RC levels can assist clinicians in the early identification and intervention of high-risk patients with breast cancer, thereby improving their prognosis.

### Electronic supplementary material

Below is the link to the electronic supplementary material.

Supplementary Material 1

## Data Availability

The datasets used and analyzed during the current study are available from the corresponding author on reasonable request.

## Abbreviations

BMI: Body mass index
HDL: High-density lipoprotein
HR: Hazard ratio
IDL: Intermediate-density lipoprotein
INSCOC: Investigation on Nutrition Status and Clinical Outcome of Common Cancers
IQR: Interquartile range
LDL: Low-density lipoproteins
OS: Overall survival
RC: Remnant cholesterol
RCS: Restricted cubic splines
VLDL: Very low-density lipoprotein
