# Supplementary material for: Remnant cholesterol is an effective biomarker for predicting survival in patients with breast cancer
Source: Nutr J. 2024 Apr 22;23:45. doi: 10.1186/s12937-024-00951-3 (PMC11034071; doi:10.1186/s12937-024-00951-3)
Supplement: Supplementary file 1 — Supplementary Material 1 [file 12937_2024_951_MOESM1_ESM.docx]

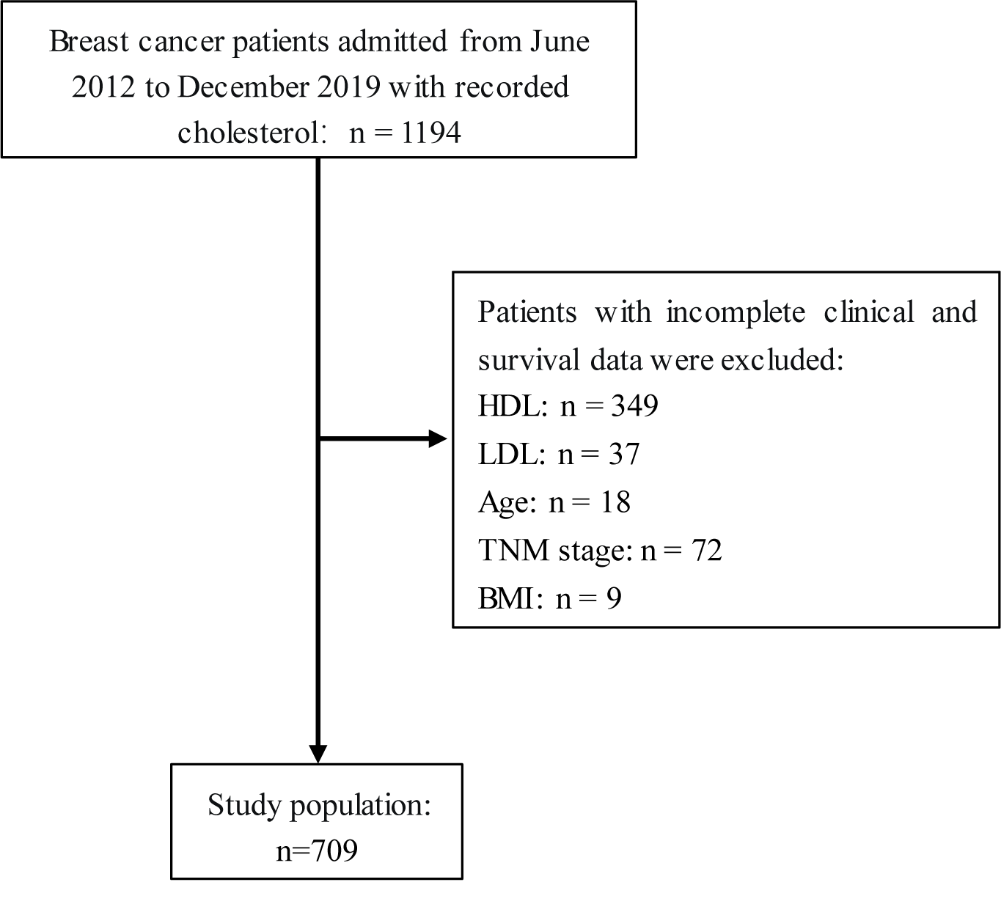


Figure S1. The flow chart.


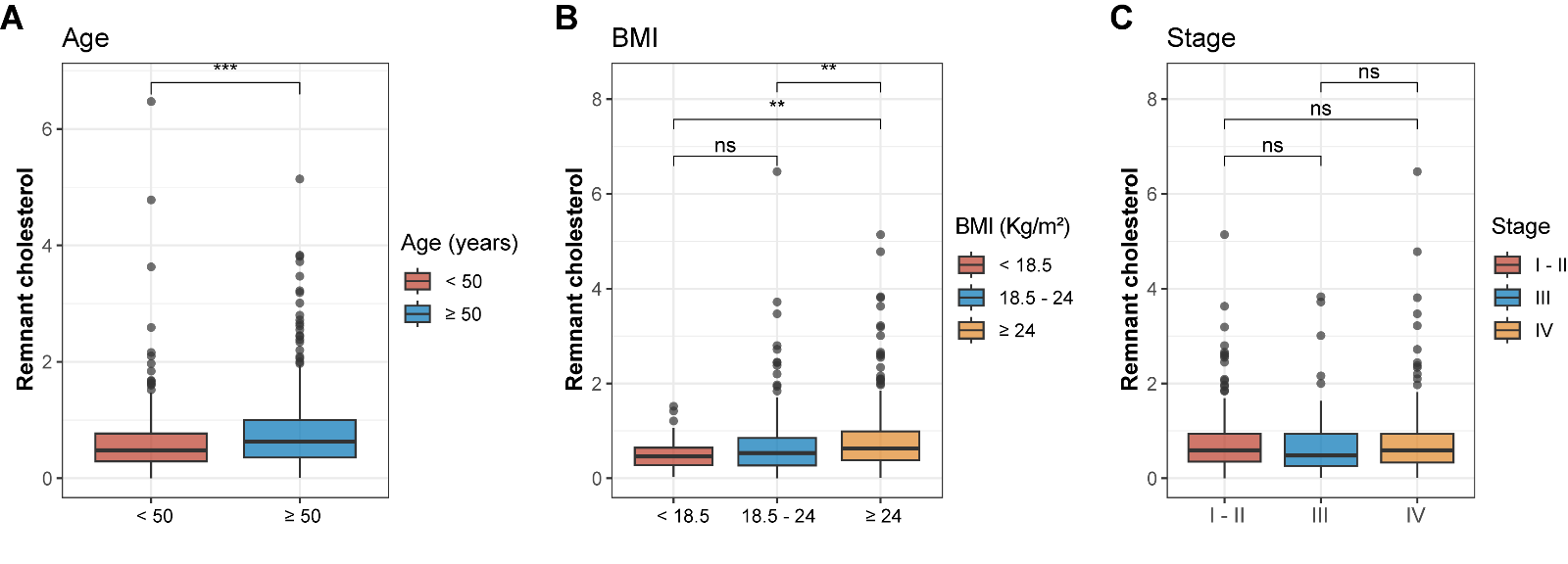


Figure S2. Remnant cholesterol in different clinicopathological subgroups. ns p-value >0.05, * p-value<0.05, **** p-value<0.001.


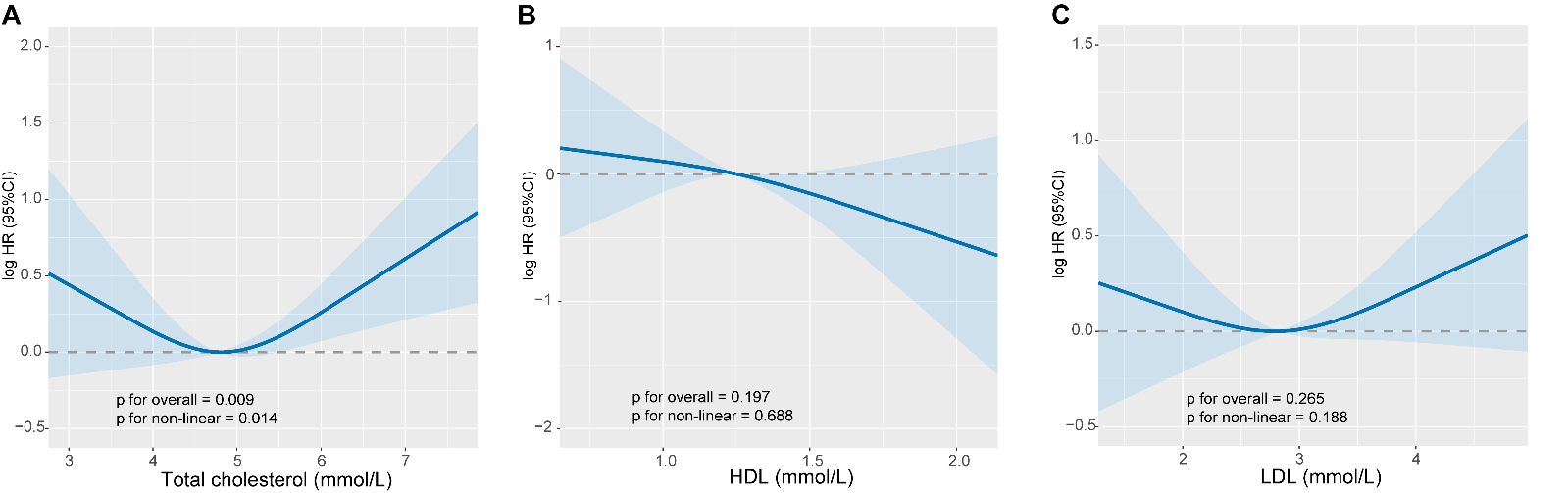


Figure S3. The relationship between total cholesterol, HDL, LDL, remnant cholesterol to total cholesterol ratio and overall survival of breast cancer patients respectively

Notes: HDL, high density lipoprotein cholesterol. LDL, low density lipoprotein cholesterol.


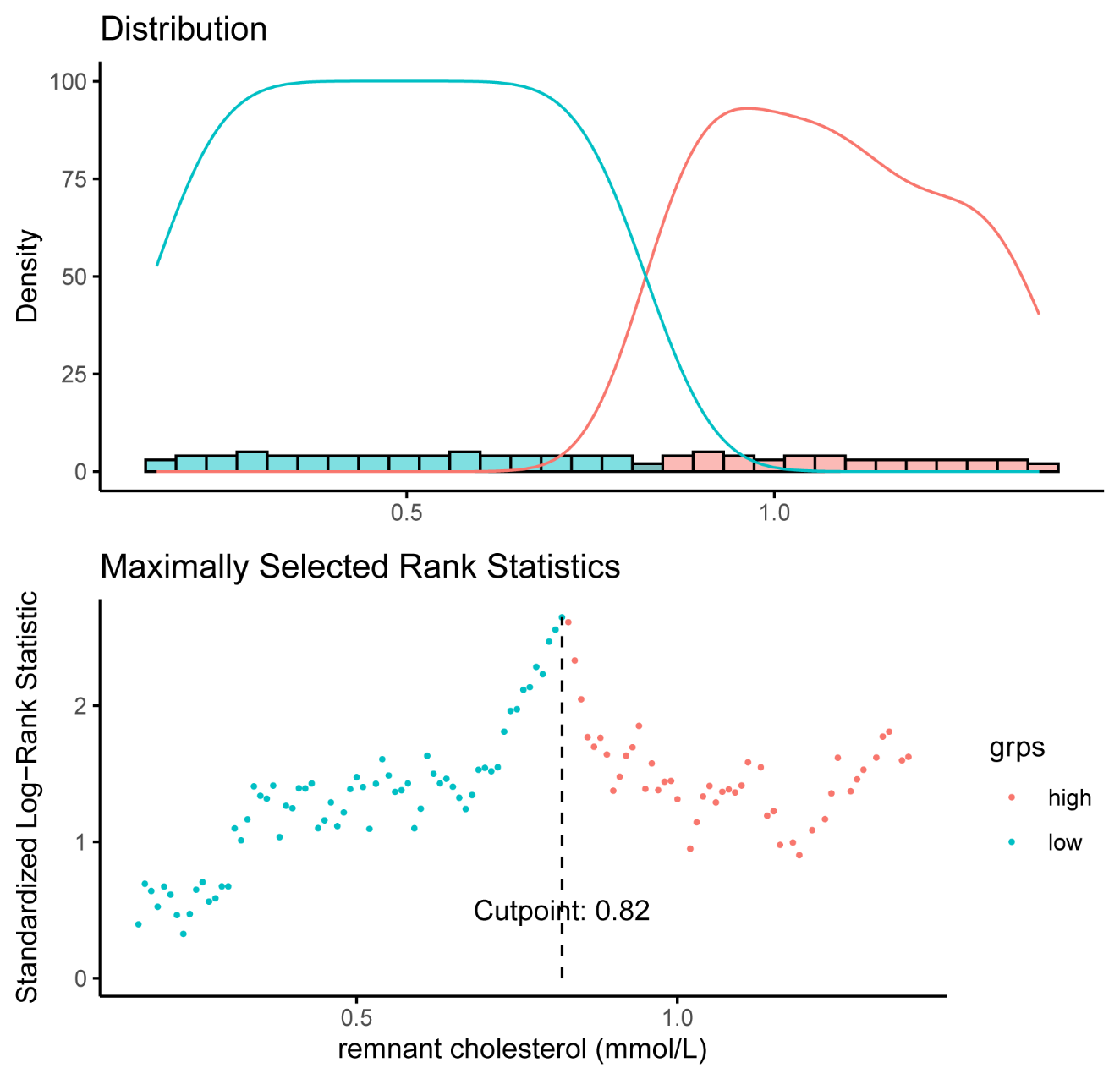


Figure S4. Cut-off value of remnant cholesterol in patients with breast cancer.


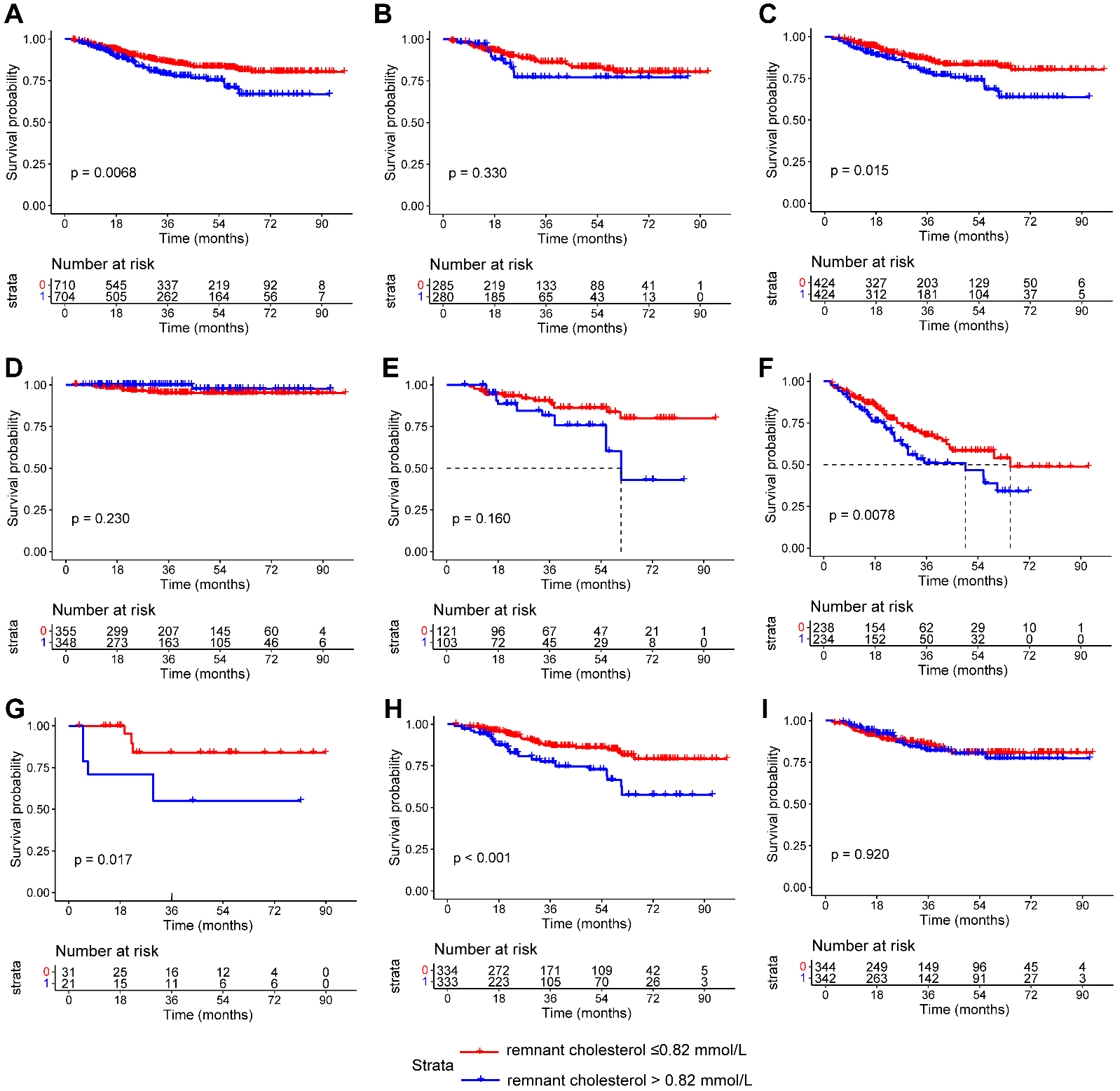


Figure S5. The Kaplan–Meier curves of breast cancer patients with low or high remnant cholesterol after IPTW. A) total population; B) premenopausal patients; C) postmenopausal patients; D) stage I–II patients; E) stage III patients; F) stage IV patients; G) underweight patients; H) normal weight patients; I) overweight patients.

Table S1. The baseline characteristics of the study population.

|  | Overall (n=709) | Tertile 1 of RC(n=237) | Tertile 2 of RC(n=236) | Tertile 3 of RC(n=236) | p |
| --- | --- | --- | --- | --- | --- |
| Age, years, median (IQR) | 52.00 [46.00, 61.00] | 50.00 [44.00, 58.00] | 51.00 [44.00, 61.00] | 55.00 [48.00, 62.00] | <0.001 |
| BMI, Kg/m2, median (IQR) | 23.88 [21.66, 26.29] | 23.23 [21.22, 25.78] | 23.80 [21.91, 26.15] | 24.44 [22.27, 26.98] | 0.001 |
| Smoking, yes, n (%) | 44 (6.2) | 10 (4.2) | 14 (5.9) | 20 (8.5) | 0.155 |
| Drinking, yes, n (%) | 13 (1.8) | 3 (1.3) | 4 (1.7) | 6 (2.5) | 0.575 |
| Diabetes, yes, n (%) | 64 (9.0) | 10 (4.2) | 24 (10.2) | 30 (12.7) | 0.004 |
| Hypertension, yes, n (%) | 110 (15.5) | 27 (11.4) | 40 (16.9) | 43 (18.2) | 0.093 |
| Coronary heart disease, yes, n (%) | 27 (3.8) | 4 (1.7) | 9 (3.8) | 14 (5.9) | 0.055 |
| Family history of tumor, yes, n (%) | 128 (18.1) | 44 (18.6) | 43 (18.2) | 41 (17.4) | 0.942 |
| Tumor stage, n (%) |  |  |  |  | 0.388 |
| I | 137 (19.3) | 44 (18.6) | 45 (19.1) | 48 (20.3) |  |
| II | 216 (30.5) | 66 (27.8) | 80 (33.9) | 70 (29.7) |  |
| III | 118 (16.6) | 50 (21.1) | 32 (13.6) | 36 (15.3) |  |
| IV | 238 (33.6) | 77 (32.5) | 79 (33.5) | 82 (34.7) |  |
| Surgery, n (%) | 84 (11.8) | 32 (13.5) | 25 (10.6) | 27 (11.4) | 0.602 |
| Chemotherapy, n (%) | 463 (65.3) | 139 (58.6) | 151 (64.0) | 173 (73.3) | 0.003 |
| Radiotherapy, n (%) | 38 (5.4) | 17 (7.2) | 11 (4.7) | 10 (4.2) | 0.309 |
| Hemoglobin, g/L, median (IQR) | 124.00 [112.00, 133.00] | 122.00 [110.00, 132.00] | 123.50 [113.00, 133.00] | 125.00 [112.00, 135.00] | 0.160 |
| White blood cells, 109/L, median (IQR) | 5.40 [4.40, 6.80] | 5.31 [4.34, 6.55] | 5.54 [4.43, 6.73] | 5.35 [4.47, 7.13] | 0.400 |
| Neutrophil, 109/L, median (IQR) | 3.20 [2.40, 4.30] | 3.18 [2.39, 4.01] | 3.18 [2.42, 4.28] | 3.31 [2.43, 4.55] | 0.374 |
| Lymphocyte, 109/L, median (IQR) | 1.56 [1.22, 2.00] | 1.52 [1.18, 1.95] | 1.65 [1.20, 2.07] | 1.55 [1.25, 1.96] | 0.221 |
| Platelets, 109/L, median (IQR) | 233.00 [186.00, 288.00] | 237.00 [189.00, 291.00] | 233.00 [190.00, 288.00] | 229.00 [183.00, 283.50] | 0.820 |
| Total protein, g/L, median (IQR) | 69.30 [65.00, 73.30] | 68.60 [64.90, 73.20] | 69.35 [64.38, 73.50] | 69.90 [65.50, 73.30] | 0.349 |
| Albumin, g/L, median (IQR) | 40.80 [37.60, 43.70] | 40.40 [37.50, 43.50] | 41.00 [37.60, 43.80] | 40.90 [37.75, 43.82] | 0.788 |
| Total bilirubin, μmol/L, median (IQR) | 9.30 [6.80, 12.30] | 9.50 [6.70, 12.04] | 9.00 [6.80, 12.30] | 9.50 [6.90, 12.43] | 0.552 |
| Direct bilirubin, μmol/L, median (IQR) | 2.70 [2.00, 3.60] | 2.90 [2.10, 3.90] | 2.60 [2.10, 3.40] | 2.60 [2.00, 3.32] | 0.046 |
| AST, U/L, median (IQR) | 22.00 [18.00, 29.00] | 21.00 [17.60, 27.00] | 21.85 [18.00, 27.00] | 23.50 [19.80, 31.70] | 0.001 |
| ALT, U/L, median (IQR) | 19.00 [13.20, 30.00] | 17.00 [12.10, 25.00] | 19.00 [13.00, 29.70] | 21.85 [15.00, 34.23] | <0.001 |
| Creatinine, μmol/L, median (IQR) | 57.00 [51.00, 63.00] | 57.20 [52.80, 63.00] | 57.00 [50.98, 63.20] | 56.40 [50.48, 62.92] | 0.303 |
| BUN, mmol/L, median (IQR) | 4.67 [3.87, 5.76] | 4.45 [3.80, 5.68] | 4.74 [3.89, 5.98] | 4.79 [3.90, 5.68] | 0.242 |
| Total cholesterol, mmol/L, median (IQR) | 4.78 [4.19, 5.53] | 4.40 [3.90, 5.13] | 4.67 [4.19, 5.31] | 5.34 [4.61, 5.98] | <0.001 |
| HDL, mmol/L, median (IQR) | 1.25 [1.06, 1.49] | 1.39 [1.20, 1.63] | 1.25 [1.07, 1.47] | 1.14 [0.99, 1.30] | <0.001 |
| LDL, mmol/L, median (IQR) | 2.83 [2.31, 3.38] | 2.76 [2.32, 3.44] | 2.79 [2.33, 3.33] | 2.90 [2.28, 3.44] | 0.804 |
| Remnant cholesterol, mmol/L, median (IQR) | 0.58 [0.32, 0.94] | 0.25 [0.14, 0.32] | 0.58 [0.48, 0.68] | 1.10 [0.94, 1.45] | <0.001 |
| Triglyceride, mmol/L, median (IQR) | 1.62 [1.14, 2.19] | 1.19 [0.89, 1.54] | 1.54 [1.19, 1.98] | 2.34 [1.73, 3.59] | <0.001 |
| Blood glucose, mmol/L, median (IQR) | 5.28 [4.84, 5.85] | 5.14 [4.79, 5.64] | 5.20 [4.76, 5.81] | 5.44 [4.95, 6.08] | <0.001 |

Notes: RC, remnant cholesterol; BMI, body mass index; AST, aspartate amino transferase; ALT, glutamate aminotransferase; BUN, blood urea nitrogen; HDL, high density lipoprotein cholesterol; LDL, low density lipoprotein cholesterol;

Tertile 1 of RC, RC < 0.41 mmol/L; Tertile 2 of RC, 0.41 mmol/L≤ RC < 0.80 mmol/L; Tertile 3 of RC, RC ≥ 0.80 mmol/L.

Table S2. The univariate and multivariate Cox analysis for the relationship between remnant cholesterol and all-cause mortality in breast cancer patients after IPTW.

|  | Crude model |  | Adjusted model |  |
| --- | --- | --- | --- | --- |
|  | HR (95%CI) | p | HR (95%CI) | p |
| Continuous | 1.38 (1.13,1.69) | 0.002 | 1.30 (1.14,1.49) | <0.001 |
| Cutoff value |  |  |  |  |
| C1 (<=0.82) | Ref |  | Ref |  |
| C2 (>0.82) | 1.66 (1.12,2.46) | 0.011 | 1.64 (1.10,2.45) | 0.016 |
| Quartiles |  |  |  |  |
| Q1 (<0.41) | Ref |  | Ref |  |
| Q2 (<0.8) | 1.07 (0.65,1.75) | 0.797 | 1.20 (0.72,2.03) | 0.485 |
| Q3 (>=0.8) | 1.61 (1.01,2.57) | 0.046 | 1.65 (1.02,2.68) | 0.041 |
| p for trend |  | 0.036 |  | 0.037 |

Notes:

Crude model: No adjusted.

Adjusted model: Adjusted for age, TNM stage, BMI, diabetes, hypertension, coronary heart disease, smoking, drinking, family history of tumor.

IPTW, Inverse probability of treatment weighting.

Table S3. The C index of the fully adjusted model and the adjusted model without RC.

|  | C index (95%CI) | p |
| --- | --- | --- |
| Adjusted model | 0.80 (0.76, 0.84) | 0.031 |
| Adjusted model - RC | 0.76 (0.72, 0.81) |  |

Notes:

Adjusted model: adjusted for age, TNM stage, BMI, diabetes, hypertension, coronary heart disease, smoking, drinking, family history of tumor;

RC, remnant cholesterol.
